# Risk factors for poor prognosis in patients with cortical laminar necrosis and establishment of their prediction model: A retrospective cohort study

**DOI:** 10.1097/MD.0000000000048871

**Published:** 2026-05-22

**Authors:** Weisen Wang, Xinyuan Zhang, Mingcheng Zhang, Binghan Li, Zhengsheng Gu, Hailing Zhang, Feng Sheng, Xiaoying Bi, Wenjia Peng

**Affiliations:** a Department of Neurology, The First Affiliated Hospital of Naval Medical University, Shanghai, China; b Department of Neurology, The 983rd Hospital of Joint Logistics Support Forces of the PLA, Tianjin, China; c Department of Radiology, The First Affiliated Hospital of Naval Medical University, Shanghai, China; d Department of Rehabilitation Medicine, Shanghai Geriatrics Center, Shanghai, China.

**Keywords:** cerebral infarction, cortical laminar necrosis, nomogram prediction model, prognostic factors

## Abstract

Cortical laminar necrosis (CLN) is a rare form of ischemic necrosis characterized by laminar damage primarily affecting the third to fifth cortical layers. CLN typically follows cerebral infarction but can also arise from various conditions. However, research on CLN is scarce, and predictive models have not yet been established, limiting clinical guidance. We aimed to analyze the risk factors for poor prognosis in patients with CLN after cerebral infarction and to construct a visual nomogram model with early predictive value. This retrospective study included 80 patients admitted to our hospital between January 2019 and December 2024. The modified Rankin Scale (mRS) was used to assess functional outcomes at 90 days after symptom onset. Clinical and imaging data were compared between patients with favorable outcomes (mRS 0–2, n = 56) and those with poor outcomes (mRS 3–6, n = 24). Independent risk factors for poor outcomes were identified through multivariable logistic regression analysis. R software was used to generate a nomogram; the model was evaluated and internally validated. Compared with the favorable outcome group, the poor outcome group had significantly higher age, admission National Institutes of Health Stroke Scale (NIHSS) score, C-reactive protein level, severity of white matter hyperintensity (WMH), and the degree of anterior cerebral artery (ACA) stenosis. Body mass index and albumin levels were significantly lower in the poor outcome than in the favorable outcome group (all *P* < .05). Admission NIHSS score (odds ratio [OR] = 1.317; 95% confidence interval [CI] 1.097–1.669; *P* = .008), WMH severity (OR = 5.273; 95% CI 1.181–28.519; *P* = .037), and degree of ACA stenosis (OR = 7.223; 95% CI 1.631–38.985; *P* = .013) were independent risk factors for poor outcome of CLN. The C-index (0.852) and calibration curve indicated that the nomogram model had high discriminative ability and accuracy. The area under the receiver operating characteristic curve was 0.867 (95% CI 0.811–0.906). Admission NIHSS score, WMH severity, and degree of ACA stenosis were independently and significantly associated with the prognosis of CLN. The nomogram model developed in this study exhibits strong predictive capability and good discriminative ability and accuracy, enhancing prognosis prediction in patients with CLN following cerebral infarction.

## 1. Introduction

Cortical laminar necrosis (CLN), first described by Sawada in 1990,^[1]^ is a form of ischemic necrosis characterized by a laminar distribution in the cortex. The pathogenesis of CLN remains unclear, but it is widely believed to be associated with insufficient cellular energy supply resulting from various causes, such as metabolic failure^[2]^ and hypoperfusion.^[3]^ In this context, the selective vulnerability of the cortex renders layers 3 to 5 more susceptible to damage.^[4]^ Although CLN is rare and mainly occurs after cerebral infarction, it can also arise from conditions such as hypoglycemic encephalopathy,^[5]^ migrainous stroke,^[6]^ encephalitis,^[7]^ and air embolism.^[8]^ In the pediatric population, hypoxic-ischemic encephalopathy constitutes the primary etiology of CLN.^[9]^ Notably, CLN appears on magnetic resonance imaging (MRI) as a “lace sign” along the cortex, with T1 hyperintensity serving as a characteristic feature and the signal intensity changing over time.^[10]^

In a previous cohort study, the incidence of CLN after cerebral infarction was reportedly 2.7% (151/5548), while the proportion of patients with poor outcomes after CLN was 22.3% (25/112).^[11]^ Moreover, higher admission National Institutes of Health Stroke Scale (NIHSS) scores and early neurological deterioration were associated with poor outcomes.^[11]^ Existing studies have consistently indicated that CLN is associated with poor clinical outcomes.^[9,11]^ Nevertheless, research on its prognosis is scarce, and prior studies have considered only a limited number of influencing factors and have not established predictive models, offering limited guidance for clinical practice. Our study addresses the limitations of prior research and fills a critical gap in this area.

Therefore, the present study aimed to improve the short-term neurological outcomes for patients with CLN. To this end, we analyzed the clinical data of patients with CLN following cerebral infarction, explored the risk factors for poor prognosis, and developed a nomogram prediction model to assist in clinical decision-making.

## 2. Materials and methods

### 2.1. Study participants

This retrospective study included patients with CLN following cerebral infarction who were admitted to the First Affiliated Hospital of Naval Medical University between January 2019 and December 2024. The inclusion criteria were as follows: diagnosis of cerebral infarction confirmed and managed in accordance with established clinical guidelines; and MRI revealing high signal intensity on T1-weighted imaging (T1WI), fluid-attenuated inversion recovery (FLAIR), and diffusion-weighted imaging (DWI) sequences along the cortex ipsilateral to the infarction. The exclusion criteria were as follows: CLN caused by mitochondrial encephalomyopathy, lactic acidosis, and stroke-like episodes or hypoglycemic encephalopathy; and incomplete clinical or imaging data. This study was approved by the Ethics Committee of the First Affiliated Hospital of Naval Medical University (approval number: CHEC2024-283). All participants provided written informed consent.

### 2.2. Clinical data collection

Demographic details gathered includedge, sex, and body mass index; medical history encompassing hypertension, diabetes, hyperlipidemia, coronary heart disease, atrial fibrillation, smoking, and alcohol consumption; clinical symptoms such as limb weakness, facial paralysis, and hemianopia; as well as laboratory indicators such as whole blood cell counts, liver and kidney function tests, blood lipid levels, coagulation function, albumin, glycated hemoglobin, C-reactive protein (CRP), and homocysteine.

The etiological subtypes of ischemic stroke were categorized based on the trial of ORG 10,172 in acute stroke treatment (TOAST) criteria,^[12]^ which included large-artery atherosclerosis, cardioembolism, small-vessel occlusion, stroke of other determined etiology, and stroke of undetermined etiology. NIHSS scores of the patients were assessed upon admission. An increase of 2 or more points in the NIHSS score within the first 7 days following admission was classified as early neurological deterioration.^[13]^

### 2.3. Imaging data collection

**Figure 1.**
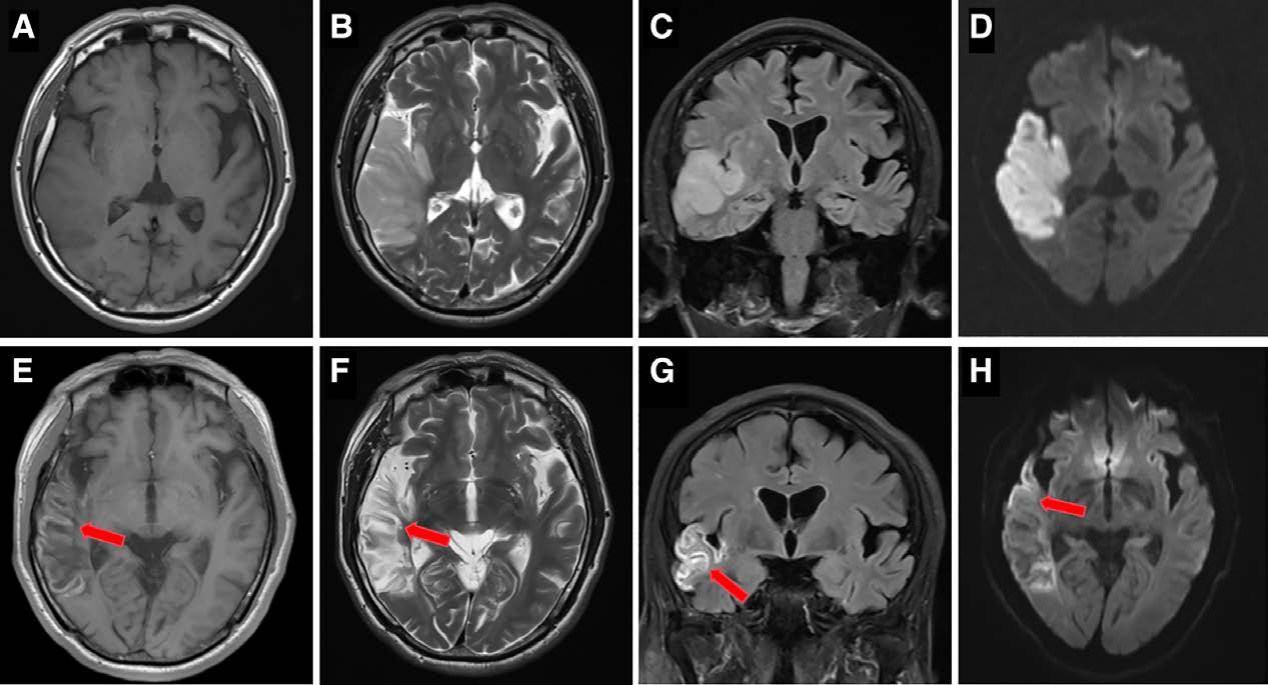
A 62-year-old man with left temporal infarction. (A–D) Images obtained at 4 days after onset, (E–H) at 35 days after onset. (A) T1WI, (B) T2WI, and (C) FLAIR do not show high signals along the cortex. (D) DWI shows laminar restricted diffusion in the right temporal lobe. (E) T1WI, (F) T2WI, (G) FLAIR, and (H) DWI from the corresponding sections show hyperintensity along the temporal gyri during follow-up which is typical of CLN. (CLN is indicated by red arrows). CLN = cortical laminar necrosis, DWI = diffusion-weighted imaging, FLAIR = fluid-attenuated inversion recovery, T1WI = T1-weighted imaging, T2WI = T2-weighted imaging.

Imaging was conducted with a standard 8-channel head matrix coil on a 3.0-T high-field MRI scanner (GE Healthcare). The scan covered the entire brain in sagittal, coronal, and axial views. The patients were instructed to use a sponge pad to restrict head movement. All patients underwent brain MRI and computed tomography angiography. Typical imaging features of CLN include high signal intensity on T1WI, FLAIR, and DWI sequences distributed along the cortex on the same side as the cerebral infarction, with signal intensity dynamically changing over time (Fig. 1). The location and number of brain lobes involved in the CLN were recorded. The assessment of vascular stenosis was conducted in accordance with the North American Symptomatic Carotid Endarterectomy Trial criteria^[14]^ and was calculated using the following formula: stenosis rate = (1 diameter of the narrowest section of the internal carotid artery/normal diameter of the distal internal carotid artery) × 100%. Stenosis was classified into categories: mild ( < 50%), moderate ( 50–70%), severe (> 70%), and occlusion (100%). White matter hyperintensity (WMH) severity was classified using the Fazekas score: 0 to 1 as mild WMH and 2 to 3 as severe WMH.^[15]^ Two qualified neurologists independently interpreted the imaging data. Disagreements were resolved by a third senior neurologists.

### 2.4. Follow-up

The modified Rankin Scale (mRS) was used to assess neurological recovery in patients with stroke. At 90 days after onset, prognostic evaluations were conducted using the mRS during outpatient appointments or through telephone follow-ups. Scores ranging from 0 to 2 were classified as favorable outcomes, while those from 3 to 6 were classified as poor outcomes.

### 2.5. Statistical analysis

The sample size was estimated to determine the independent association between anterior cerebral artery (ACA) stenosis and poor prognosis using multivariate logistic regression analysis. The calculation was based on the method proposed by Hsieh, Bloch, and Larsen [Hsieh, F. Y., Bloch, D. A., & Larsen, M. D. (1998). A simple method of sample size calculation for linear and logistic regression. Statistics in Medicine, 17 (14), 1623–1634]. We assumed a 2-sided Type I error rate (alpha) of 0.05 and a statistical power (1-beta) of 80%. Based on preliminary data and clinical assumptions, the prevalence of ACA stenosis in the study population was estimated at 20%, and the overall incidence of poor prognosis was estimated at 30%. To detect a minimum odds ratio(OR) of 6.0, while adjusting for other covariates (assuming a squared multiple correlation, *R*^2^, of 0.10 between the predictor and other covariates), the minimum required sample size was calculated to be 81 patients.

Statistical analysis was conducted using SPSS software (version 25.0; IBM). The Kolmogorov–Smirnov test was employed to evaluate the normality of all measurement data. Data exhibiting a normal distribution were presented as mean ± standard deviation, and group comparisons were carried out using independent sample *t*-tests or analysis of variance. For data not following a normal distribution, results were reported as medians and interquartile ranges, with comparisons performed using the Mann–Whitney *U* test. Categorical data were summarized as frequencies and percentages, with comparisons performed via the χ^2^ or Fisher exact test. Variables showing a *P*-value < .05 in univariate logistic regression analysis, as well as clinically significant variables, were further analyzed to compute the variance inflation factor (VIF) using Python 3.7. Variables with a VIF exceeding 10 were regarded as having substantial collinearity with other variables. Independent variables (VIF ≤ 10) were selected for multivariate logistic regression analysis, treating prognosis as the dependent variable while calculating the OR and 95% confidence interval (CI). A statistical significance threshold was set at *P* < .05.

A nomogram predictive model for the prognosis of CLN was constructed using R (version 3.6.2; R Foundation for Statistical Computing, Vienna). The C-index was used to assess the discrimination of the predictive model. Internal validation was conducted via the bootstrap method, incorporating 5000 resamples, and calibration curves were generated to determine the predictive model’s accuracy. Additionally, receiver operating characteristic (ROC) curves were constructed to evaluate the efficacy of the nomogram.

## 3. Results

### 3.1. Demographic information

**Figure 2.**
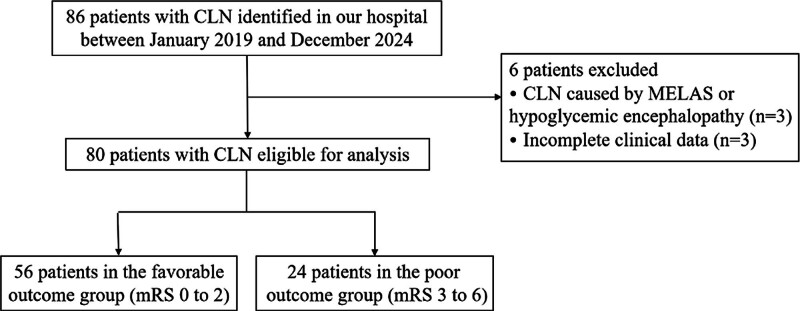
Flow chart of the study. CLN = cortical laminar necrosis, mRS = modified Rankin scale.

Overall, 86 patients met the diagnostic criteria for CLN. After excluding 3 cases due to other causes (2 mitochondrial encephalomyopathy, lactic acidosis, and stroke-like episodes and 1 hypoglycemic encephalopathy) and 3 cases due to missing imaging data, 80 patients were included in the study (Fig. 2). The patients were divided into the favorable outcome (n = 56, median age: 62.50 [53.00, 70.00] years, male: 69.6%) and poor outcome groups (n = 24, median age: 70.00 [63.75, 73.00] years, male: 62.5%).

### 3.2. Clinical characteristics and imaging features of CLN

The age, admission NIHSS score, and CRP levels were significantly higher in the poor outcome group than in the favorable outcome group, whereas body mass index and albumin levels were significantly lower (all *P* < .05; Table 1).

**Table 1:** Clinical characteristics of the 2 groups.

Variable	All (n = 80)	Favorable Outcome (n = 56)	Poor Outcome (n = 24)	*t* or *χ*^2^ or z	*P* value
Demographic information					
Age (years), median [IQR]	65.00 [56.00, 71.00]	62.50 [53.00, 70.00]	70.00 [63.75, 73.00]	2.129	.006*
Sex (male), n (%)	54 (67.5)	39 (69.6)	15 (62.5)	0.391	.532
BMI (kg/m^2^), mean ± SD	24.42 ± 3.02	24.87 ± 3.05	23.38 ± 2.73	−2.123	.042*
Risk factors					
Hypertension, n (%)	53 (66.3)	35 (62.5)	18 (75.0)	1.174	.409
Diabetes, n (%)	22 (27.5)	14 (25.0)	8 (33.3)	0.585	.623
Hyperlipidemia, n (%)	24 (30.0)	18 (31.6)	6 (27.3)	0.139	.709
Coronary heart disease, n (%)	7 (8.8)	7 (12.5)	0 (0.0)	3.288	.167
Atrial fibrillation, n (%)	15 (18.8)	10 (17.9)	5 (20.8)	0.098	.755
Smoking, n (%)	33 (41.3)	23 (41.1)	10 (41.7)	0.246	.620
Alcohol consumption, n (%)	27 (33.8)	19 (33.9)	8 (33.3)	0.266	.606
Clinical manifestations and scores					
Limb weakness, n (%)	33 (41.3)	23 (41.1)	10 (41.7)	0.002	.960
Facial palsy, n (%)	16 (20.0)	11 (19.6)	4 (16.7*)	0.098	.755
Hemianopia, n (%)	9 (11.3)	6 (10.7)	3 (12.5)	0.054	.817
Laboratory indicators					
Albumin, median [IQR]	40.90 [37.75, 44.00]	41.50 [39.00, 44.00]	38.00 [36.00, 42.25]	−2.886	.028*
CRP, median [IQR]	4.44 [2.34, 12.70]	4.31 [2.07, 9.14]	7.84 [4.16, 13.20]	2.950	.048*
Stroke etiologic subtypes					
LAA, n (%)	43 (53.8)	26 (46.4)	17 (70.8)	4.025	.088
CE, n (%)	10 (12.5)	6 (10.7)	4 (16.7)		
SAO, n (%)	4 (5)	4 (7.1)	0 (0)		
SOE, n (%)	16 (20)	15 (26.8)	1 (4.2)		
SUE, n (%)	7 (8.8)	5 (8.9)	2 (8.3)		
Admission NIHSS score, median [IQR]	2.00 [0.00, 4.00]	1.00 [0.00, 2.25]	4.50 [2.00, 7.00]	3.650	< .001*
END, n (%)	6 (7.5)	2 (3.6)	4 (16.7)	2.48	.115

BMI = body mass index, CE = cardioembolism, CRP = C-reactive protein, END = early neurologic deterioration, IQR = interquartile range, LAA = large-artery atherosclerosis, n = number of patients, NIHSS = National institutes of health stroke scale, SAO = small-vessel occlusion, SD = standard deviation, SOE = stroke of other determined etiology, SUE = stroke of undetermined etiology.

* *P* < .05.

The severity of cerebral WMH and degree of ACA stenosis were significantly greater in the poor outcome group than in the favorable group (both *P* < .05; Table 2). No differences were observed between the 2 groups regarding the distribution and number of affected brain lobes or the severity of stenosis in arteries other than the ACA.

**Table 2:** Imaging characteristics of the 2 groups.

Variable	All (n = 80	Favorable outcome (n = 56	Poor outcome (n = 24	*t* or χ^2^ or z	*P* value
Lesion location					
Frontal lobe, n (%	35 (43.8)	24 (42.9)	11 (45.8)	0.060	1
Temporal lobe, n (%	38 (47.5)	26 (46.4)	12 (50)	0.086	.961
Parietal lobe, n (%	43 (53.8)	29 (51.8)	14 (58.3)	0.290	.769
Occipital lobe, n (%	27 (33.8)	18 (32.1)	9 (37.5)	0.216	.836
Insular lobe, n (%	5 (6.3)	2 (3.6)	3 (12.5)	2.286	.313
Number of brain lobes affected, n (%)					
Single lobe	66 (82.5)	44 (78.6)	22 (91.7)	1.995	.158
Multiple lobes	14 (17.5)	12 (21.4)	2 (8.3)		
Degree of cervical artery stenosis, n (%)					
Normal	43 (53.75)	30 (53.6)	13 (54.2)	3.021	.388
Mild stenosis	14 (17.5)	12 (21.4)	2 (8.3)		
Moderate stenosis	4 (5)	3 (5.4)	1 (4.2)		
Severe stenosis-Occlusion	19 (23.75)	11 (19.6)	8 (33.3)		
Degree of anterior cerebral artery stenosis, n (%)					
Normal	62 (77.5)	49 (87.5)	13 (54.2)	8.381	.003*
Mild stenosis	6 (7.5)	2 (3.6)	4 (16.7)		
Moderate stenosis	3 (3.75)	1 (1.8)	2 (8.3)		
Severe stenosis-Occlusion	9 (11.25)	4 (7.1)	5 (20.8)		
Degree of middle cerebral artery stenosis , n (%)					
Normal	42 (52.5)	27 (48.2)	15 (62.5)	2.334	.506
Mild stenosis	5 (6.25)	3 (5.4)	2 (8.3)		
Moderate stenosis	7 (8.75)	5 (8.9)	2 (8.3)		
Severe stenosis-Occlusion	26 (32.5)	21 (37.5)	5 (20.8)		
Degree of posterior cerebral artery stenosis n (%)					
Normal	71 (88.75)	48 (85.7)	23 (95.8)	3.575	.167
Mild stenosis	2 (2.5)	1 (1.8)	1 (4.2)		
Moderate stenosis	0 (0)	0 (0)	0 (0)		
Severe stenosis-Occlusion	7 (8.75)	7 (12.5)	0 (0)		
Degree of vertebral artery stenosis n (%)					
Normal	72 (90)	48 (85.7)	24 (100)	3.810	.051
Mild stenosis	0 (0)	0 (0)	0 (0)		
Moderate stenosis	0 (0)	0 (0)	0 (0		
Severe stenosis-Occlusion	8 (10)	8 (14.3)	0 (0)		
WMH grade , n (%)					
Mild WMH (0–1)	48 (60.0	41 (73.2)	7 (29.2)	7.235	.001*
Severe WMH (2–3)	32 (40.0	15 (26.8	17 (70.8)		

WMH = white matter hyperintensity, n = number of patients.

* *P* < .05.

### 3.3. Independent risk factors for poor outcome in patients with CLN

In the univariate logistic regression analysis, variables that yielded a *P* value  < .05 included age, admission NIHSS score, albumin level, severity of WMH, and degree of ACA stenosis (Table 3). Collinearity among these variables was assessed, and no significant relationship was observed. Subsequently, multivariate logistic regression analysis demonstrated that the admission NIHSS score (OR = 1.317; 95% CI 1.097–1.669; *P* = .008), severity of WMH (OR = 5.273; 95% CI 1.181–28.519; *P* = .037), and degree of ACA stenosis (OR = 7.223; 95% CI 1.631–38.985; *P* = .013) were independent risk factors for poor outcomes in patients with CLN (Table 4).

**Table 3:** Univariate logistic regression analysis for poor outcomes of patients with CLN.

	OR	95% CI	*P*-value
Age	1.053	1.006–1.109	.038*
BMI	0.840	0.692–0.997	.058
Admission NIHSS	1.309	1.132–1.565	.001*
Albumin	0.854	0.751–0.956	.009*
CRP	1.047	1.017–1.115	.145
Severity of WMH	6.638	2.384–20.276	< .001*
Degree of ACA stenosis	5.923	1.962–19.161	.002*

ACA = anterior cerebral artery, BMI = body mass index, CI = confidence interval, CLN = cortical laminar necrosis, CRP = C-reactive protein, NIHSS = National institutes of health stroke scale, OR = odds ratio, WMH = white matter hyperintensity.

* *P* < .05.

**Table 4:** Multivariate logistic regression analysis for poor outcomes of patients with CLN.

	OR	95% CI	*P* value
Age	1.011	0.929–1.096	.783
Admission NIHSS	1.317	1.097–1.669	.008*
Albumin	0.914	0.746–1.100	.357
severity of WMH	5.273	1.181–28.519	.037*
Degree of ACA stenosis	7.223	1.631–38.985	.013*

ACA = anterior cerebral artery, CI = confidence interval, CLN = cortical laminar necrosis, NIHSS = National institutes of health stroke scale, OR = odds ratio, WMH = white matter hyperintensity.

* *P* < .05.

### 3.4. Nomogram predictive model for poor CLN prognosis

**Figure 3.**
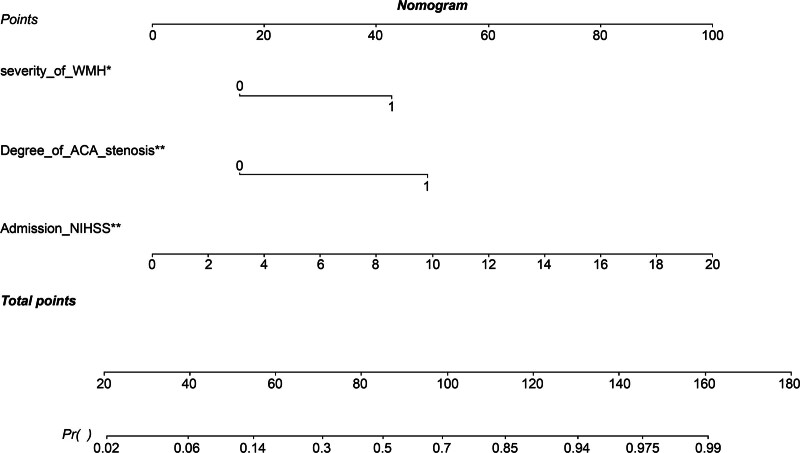
Nomogram for predicting poor prognosis in patients with cortical laminar necrosis. A single asterisk (*) indicates a *P* value < .05, and double asterisks (**) indicate a *P* value < .001. ACA = anterior cerebral artery, NIHSS = National Institutes of Health Stroke Scale, WMH = white matter hyperintensity.

**Figure 4.**
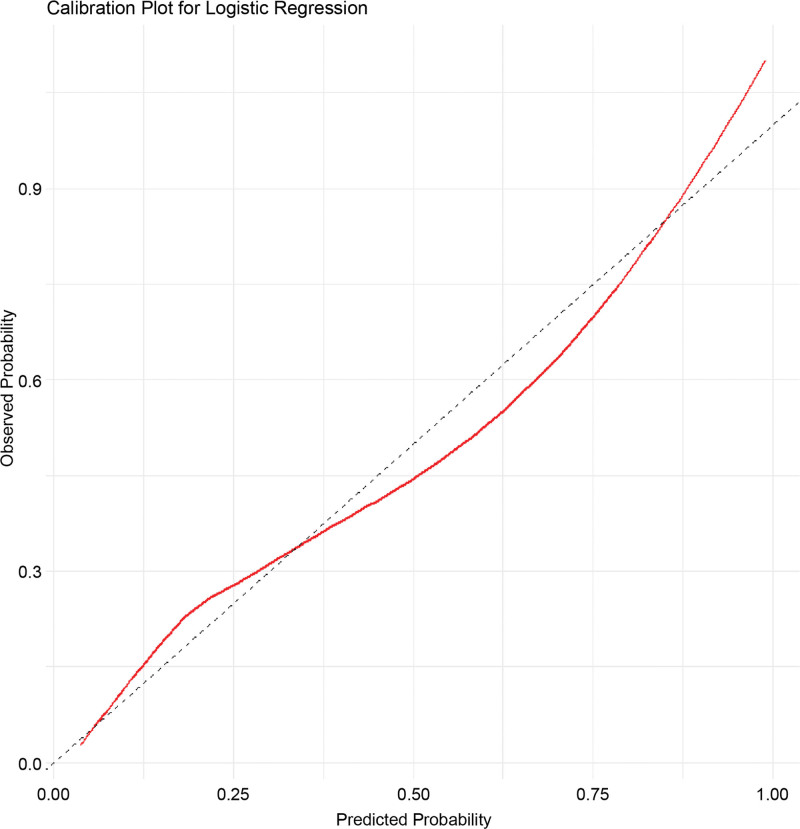
Calibration curves for the nomogram used for predicting poor prognosis in patients with cortical laminar necrosis. The red line represents the C-index of the model.

**Figure 5.**
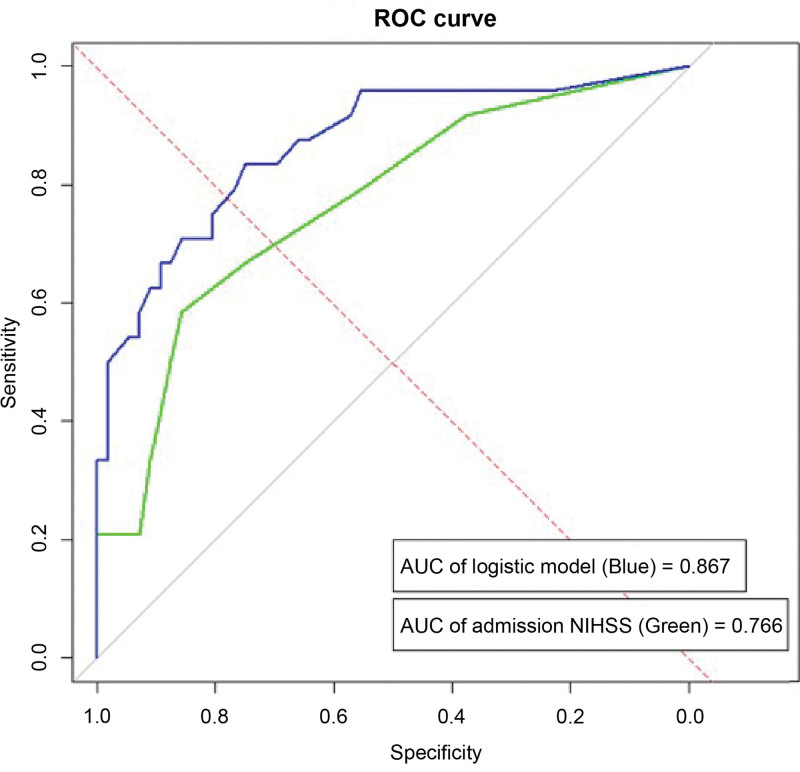
Receiver operating characteristic curve of admission NIHSS score alone(the green line) and logistic model combined admission NIHSS, severity of WMH and degree of ACA stenosis(the blue line). ACA = anterior cerebral artery, NIHSS = National Institutes of Health Stroke Scale, ROC = receiver operating characteristic, WMH = white matter hyperintensity..

A nomogram predictive model for poor prognosis of CLN was established based on 3 factors: admission NIHSS score, severity of WMH, and degree of ACA stenosis (Fig. 3). The C-index of the model was 0.852, suggesting that the nomogram model has good discriminative ability. The calibration curve of the model aligned with the ideal model curve, indicating high accuracy (Fig. 4). The ROC curve exhibited an area under the curve (AUC) of 0.867 (95% CI: 0.811–0.906) and the optimal cutoff value was 0.230 (sensitivity 83.8%, specificity 75.3%, YI 0.585), suggesting that the nomogram model exhibits high efficacy in predicting poor prognosis of CLN (Fig. 5). In addition, the admission NIHSS score alone showed lower predictive power, with an AUC of 0.766 (95% CI 0.742–0.798). The optimal cutoff value was 2.537 (sensitivity 66.3%, specificity 75.2%, YI 0.421) (Table 5).

**Table 5:** ROC curve analysis of admission NIHSS and logistic model combined admission NIHSS, severity of WMH and degree of ACA stenosis.

	AUC	95% CI	Cutoff value	Sensitivity %	Specificity %	YI
Admission NIHSS	0.766	0.742–0.798	2.537	66.3	75.2	0.421
Logistic Model	0.867	0.811–0.906	0.230	83.8	75.3	0.585

ACA = anterior cerebral artery, AUC = Area under the curve, CI = confidence interval, NIHSS = National institutes of health stroke scale, ROC = Receiver operating characteristic, WMH = white matter hyperintensity, YI = Youden’s index.

* *P* < .05.

## 4. Discussion

The present study revealed that admission NIHSS score, severity of WMH, and the degree of ACA stenosis were independent risk factors for poor outcomes in patients with CLN. The nomogram model based on these 3 factors demonstrated good predictive ability, discrimination, and accuracy, enhancing the efficacy of prognosis prediction for patients with CLN following cerebral infarction.

The chronological order of signal alterations across different MRI sequences reflects the underlying pathophysiological progression. At the onset of cellular death, DWI typically demonstrates restricted diffusion.^[16]^ According to prior reports, these DWI changes emerge within 48 hours and persist for at least 5 to 7 days following the acute event.^[2,3,9]^ A notable imaging characteristic linked to the deposition and degradation of substantial quantities of protein after the necrosis of both neurons and glial cells is the elevated T1WI signal observed along the cortex, as opposed to being related to hemorrhage.^[17,18]^ A cross-sectional study has indicated that a cortical high signal on T1WI appears approximately 2 weeks after cerebral infarction, peaks at 1 to 3 months, and can persist for up to 2 years.^[3,10]^ In the subacute to chronic phase, FLAIR hyperintensity becomes apparent, reflecting reactive gliosis in affected areas. Consistent with previous studies, our findings demonstrated a similar pattern of signal evolution over time. The combined assessment of DWI, T1WI, and FLAIR sequences facilitated the diagnosis of CLN in our cohort.

In our study, most patients (70%) had an mRS score of 0 to 2 at 90 days, which is consistent with previous research findings.^[11]^ In the poor outcome group of our study, albumin levels, indicative of nutritional status, were lower, and CRP levels, indicative of inflammation, were higher than those in the favorable outcome group. Notably, the NIHSS score at admission was higher in the poor outcome group and was significantly associated with poor outcomes. Moreover, our comparison of clinical symptoms, such as limb weakness, facial paralysis, and hemianopia, between the 2 groups revealed no significant differences. To better identify factors influencing prognosis, a more detailed analysis of the NIHSS subitems would be beneficial. The ROC curve showed that admission NIHSS score was statistically predictive of the functional outcome of CLN, with an AUC of 0.766 and an optimal cutoff value of 2.537.

Given that most patients with CLN with poor outcomes in our study had large-artery atherosclerosis according to the TOAST criteria (70.8%), we speculated that large-vessel occlusion and collateral circulation failure may contribute to the poor outcome. Therefore, we further analyzed the degree of stenosis in the large intracranial and extracranial vessels and found that ACA stenosis was associated with poor outcomes. The superficial cortical branches of the ACA mainly supply the medial surface of the anterior 3-fourths of the cerebral hemisphere and the dorsal 1-fourth of the frontal and parietal lobes. In contrast, the deep cortical branches supply the cingulate gyrus and corpus callosum. The deep penetrating branches mainly supply the anterior limb of the internal capsule, caudate nucleus, anterior part of the lentiform nucleus, and hypothalamus.^[19]^ Notably, patients with ACA stenosis exhibit cognitive dysfunction, especially in the domain of executive function, and the greater the degree of stenosis, the poorer the collateral circulation and cognitive function.^[20]^ Executive dysfunction following stroke has been increasingly recognized as a critical determinant of functional recovery. Recent evidence indicates that executive dysfunction significantly predicts motor inconsistency (characterized by heightened fluctuations in motor performance across repeated attempts) which directly compromises the capacity to engage effectively in rehabilitation tasks.^[21]^ Executive function has been identified as a strong and independent predictor of functional recovery following stroke rehabilitation.^[22]^ Longitudinal studies further confirm a moderate-to-strong negative correlation between executive function and functional outcomes, including mRS scores, throughout the recovery period.^[23]^ Therefore, we hypothesize that executive function impairment related to ACA stenosis may hinder motor learning, impede active participation in rehabilitation, and compromise adherence to treatment guidelines, ultimately contributing to poor functional recovery.

WMH is one of the imaging markers of cerebral small-vessel disease. The main pathophysiological mechanisms involve microvascular changes due to reduced cerebral blood flow, blood–brain barrier disruption, and neurovascular unit damage.^[24]^ Our findings indicate that an elevated WMH load could influence the functional recovery of CLN. The link between WMH load and poor outcomes may be partially due to WMH-related microcirculatory changes, which can lead to pericyte-mediated blood–brain barrier damage,^[25]^ impairment in maintaining or enhancing cerebral blood flow,^[26]^ or decreased cerebral angiogenesis.^[27]^

In summary, the present study developed a nomogram prediction model based on the independent risk factors identified for poor prognosis of CLN. The model demonstrated high predictive accuracy, with an AUC of 0.867, a sensitivity of 83.8%, and a specificity of 75.3%; its efficacy was superior to that of single-factor detection. This model can be used for individualized assessment of the risk of poor prognosis in CLN, facilitating the development of appropriate follow-up plans for different risk groups, which is beneficial for early detection and intervention.

Nevertheless, this study has several limitations. First, as a single-center retrospective study without long-term follow-up, our findings require validation through larger, multi-center studies. Prospective cohort designs are needed to better control for potential confounders, including treatment modalities and rehabilitation intensity. Second, cognitive function was not assessed in our cohort. Given that CLN predominantly involves the frontal and temporal lobes, such involvement may lead to cognitive impairment, which could in turn influence patient prognosis. Third, infarct size was estimated using the number of affected lobes (a semiquantitative approach) rather than precise volumetric measurements. As such, future studies employing quantitative volumetric methods are warranted to validate our findings. Finally, although our sample size (n = 80) approximated the calculated requirement (n = 81), the number of outcome events was relatively small. This creates a potential risk of overfitting in the multivariable model. We attempted to mitigate this by limiting the number of predictors and using bootstrap validation, but these findings should be interpreted with caution and validated in larger, external cohorts.

## 5. Conclusions

Admission NIHSS score, WMH severity, and degree of ACA stenosis are independent risk factors for poor prognosis in patients with CLN following cerebral infarction. The visual nomogram prediction model constructed based on these risk factors exhibits good predictive ability, enabling the intuitive analysis of each risk factor. This model enables healthcare professionals to rapidly and easily assess the probability of poor prognosis in patients with CLN, providing a reference for developing individualized clinical plans.

## Acknowledgments

We fully acknowledge all the participants who gave their precious time and wish to thank all members for their hard work and patience. We would like to thank Editage (www.editage.com) for English language editing.

## Author contributions

**Conceptualization:** Xiaoying Bi, Wenjia Peng.

**Data curation:** Weisen Wang, Xinyuan Zhang, Mingcheng Zhang.

**Investigation:** Xinyuan Zhang.

**Formal analysis:** Binghan Li.

**Project administration:** Hailing Zhang.

**Resources:** Zhengsheng Gu, Hailing Zhang.

**Software:** Binghan Li, Zhengsheng Gu.

**Visualization:** Feng Sheng.

**Writing – original draft:** Weisen Wang, Xinyuan Zhang, Mingcheng Zhang.

**Writing – review & editing:** Xiaoying Bi, Wenjia Peng.
